# Smartphone-Assisted Plasmonic Nanosensor for Visual and Specific Sensing of Toxic Cyanide Ions by β−Cyclodextrin Templated Gold-Rich/Silver Bimetallic Alloy Nanoparticles

**DOI:** 10.3390/ma18071604

**Published:** 2025-04-02

**Authors:** Nguyen Nam Phuong Truong, Ramar Rajamanikandan, Kandasamy Sasikumar, Heongkyu Ju

**Affiliations:** 1 Department of Physics and Semiconductor Science, Gachon University, Seongnam-si 13120, Republic of Koreasasiphy2022@gachon.ac.kr (K.S.); 2 Gachon Bionano Research Institute, Gachon University, Seongnam-si 13120, Republic of Korea

**Keywords:** cyanide ion, alloy nanoparticles, Ag and Au alloys, smartphone, plasmons, colorimetry

## Abstract

As cyanide ion (CN^−^), an ecologically harmful pollutant, has received incessant attention with growing industrialization on a global scale, the capability of on-site monitoring of CN^−^ contamination becomes increasingly crucial. In this work, we have fabricated a simplistic plasmonic-sensing platform for CN^−^, which can be combined with the human naked eye for visual monitoring. The main sensor part consisted of β-Cyclodextrin (β−CD)-decorated gold-rich silver bimetallic alloy nanoparticles (β−CD-Ag/Au-rich alloy NPs), while a sensing analysis was performed by a spectrophotometer or smartphone, where optical data gathered by its camera were analyzed by RGB color sensing. Upon the introduction of various CN^−^ quantities into β−CD-Ag/Au-rich alloy NPs, the spectral peak of the surface plasmon resonance (SPR) shifted from 488 nm to 496 nm. This redshift indicated a strong etching reaction between alloy NPs and CN^−^, demonstrating a ultrahigh detection sensitivity, i.e., a limit of detection (LOD) of 0.24 nM. During the formation of metal-cyano complexes in the CN^−^-induced etching response of β−CD-Ag/Au-rich alloy NPs, we observed a naked-eye discernible color change from brownish-red to colorless, allowing for naked-eye monitoring. The smartphone could also analyze the colorimetric response for such an etching process via RGB color sensing, demonstrating a LOD of 1.35 nM, being still less than the maximum concentration (1.91 nM) in drinking water, which is allowable by the World Health Organization (WHO). The straightforwardness and very high sensitivity of the proposed technique for CN^−^ detection using alloy nanoparticles with a smartphone may hold promise for simplistic, affordable in-field examinations of CN⁻ in water.

## 1. Introduction

The cyanide ion, CN^−^ is one of the most harmful elements present in water sources, contaminating ecological systems, including the human body. It is known to cause malfunctions in human cell respiration, which may even result in unconsciousness and death [1,2,3]. While CN^−^ is found in nature in a variety of forms [4,5], metallurgy, electroplating, and organic polymer industries continue to produce CN^−^, poisoning water resources in the environment [4]. Owing to its harmful impact, the World Health Organization (WHO) allowed the maximum dose in drinking water to be set as 1.9 mM [6,7]. Therefore, because of CN^−^’s toxicity, the development of cheaper sensors without compromising sensitivity for the quantitative detection of CN^−^ has been demanded.

At present, high-performance liquid chromatography (HPLC) is most frequently used, as it is a trustworthy analysis instrument with ultrahigh sensitivity that can be used for determining the amount of CN^−^ that exists in food and environmental water [8,9]. However, this has been subject to non-straightforward lengthy operations that require highly trained experts and large amounts of toxic organic solvents, as well as suffering from high operational costs and chromatographic column degradation. Therefore, it is essential to develop a simpler, cost-effective analysis strategy without compromising sufficient sensitivity. Frequently, colorimetric techniques can lend themselves to resolving these problems since they are inexpensive, easy to use, and produce analysis results quickly while securing sufficient sensitivity [3,5]. In particular, the plasmonic nanostructure-based colorimetric approach has gained increasing attention due to its advantages of label-free real-time detection, as well as a fast response time, ultrahigh sensitivity, and a capability of multiplexing/device miniaturization, as proven in various applications such as medical diagnostics [10,11], food safety [12,13], and environmental monitoring [14,15]. The major properties of the plasmonic approaches that yield such advantages inherently arise from an enhancement of local electric fields near metal nanostructures [16,17,18]. These enhanced local fields ramp up the light–matter interaction without destroying the analyte, as demonstrated in various applications, including surface-enhanced spectroscopy [19,20,21]

Nanoparticles (NPs) of either gold (Au) or silver (Ag) are commonly used as plasmonic nanoprobes for CN^−^ sensing because, when exposed to oxygen, the ions easily form metal-cyano complexes via the Elsner reaction [1,5]. Several organic molecules combined with Au/Ag NPs have been previously reported for the fluorescence-based sensing of CN^−^ with a sensitivity higher than colorimetric assays [11,14]. The challenges often faced in developing fluorescence-based approaches usually arise from organic fluorophores, which are toxic molecules that are only dissolvable in organic solvents, thereby restricting practical use. Recently, Au/Ag bimetallic core-shell NPs have been utilized to identify CN^−^, supporting easy, rapid detection with both high sensitivity and specificity [5,15,22]. The plasmonic spectral band of Au-Ag bimetallic core–shell NPs could be sensitively altered by slightly varying the core–shell dimensional ratio [23,24,25]. CN^−^ is expected to form a metal cyanide complex, thus altering the core–shell dimensional ratio via the Elsner reaction with oxygen dissolved in water. It is seen that core–shell NPs such as Au@Ag or Ag@Au outperform the monometallic Au or Ag NPs, benefitting effective visual sensing of CN^−^ with increased visibility of a color change [1,14,22,26]. Such color changes can then be analyzed by digital imaging devices or spectrophotometric instruments, as currently implemented in colorimetry. Portable devices such as smartphones can also analyze color changes, boosting the capability of real-time in situ monitoring [27,28,29].

It is known that the equilibrium constant of the chemical response between Au and CN^−^ (K = 4.4 × 10^28^) is larger than between Ag and CN^−^ (K = 9.4 × 10^24^) [26]. This led us to use β−CD-Ag/Au-rich alloy NPs for which an unmodified β−CD could be utilized as both a surface stabilizer and a reducing agent. Such alloy NPs were used as plasmonic nanoprobes for detecting CN^−^ with a smartphone-integrated colorimetric sensor. The aforementioned CN^−^ induced the formation of metal-cyano complexes that influenced the plasmonic spectral band and offered the basis for colorimetric sensing of CN^−^. The colorimetric approach with these alloy NPs offered a limit of detection of 0.24 nM CN^−^, indicating an excellent sensitivity when using an absorbance spectroscopy technique. Such a colorimetric response can also be acquired by a smartphone (cameras and RGB color recognition ability). In particular, the smartphone-based assay was found to support a user-friendly, portable, and reliable (specific) quantification of CN^−^ with a limit of detection (LOD) of 1.35 nM, holding great promise for straightforward, cost-effective, very rapid, and sufficiently sensitive sensing of CN^−^ in environmental water sources.

## 2. Experimental Procedures

### 2.1. Materials and Instruments

β−CD, sodium hydroxide (NaOH), silver nitrate (AgNO_3_), and tetrachloroauric acid (HAuCl_4_) were procured from Sigma Aldrich, Saint Louis, MO, USA, and used as received. NaCN was bought from Loba Chemie Pvt. Ltd., Mumbai, India. All the reagents used in this study are of minimum analytical reagent grade quality. Every aqueous solution was made using ultrapure water that was purchased from the Republic of Korea’s Taeyoung Pure Water Series. Absorbance titration investigations at ambient conditions were performed with an absorption spectrophotometer (JASCO 750, Tokyo, Japan). Fourier transmittance infrared (FT-IR) spectra of β-CD and β-CD-Ag/Au-rich alloy NPs were analyzed by an infrared spectrometer (FT-IR-4600, JASCO, Tokyo, Japan) via the ATR mode. The morphology of the β-CD-Ag/Au-rich alloy NPs was monitored by a high-resolution transmission electron micrographs (HR-TEM) machine with a 200 kV working voltage (TECNAI G2 F30, FEI, Hillsboro, OR, USA). Using a device (Multilaβ-2000, Thermo Scientific, Waltham, MA, USA) outfitted with a monochromatic AlK X-ray source (1486.6 eV) for excitation, X-ray photoelectron spectroscopy (XPS) studies were conducted.

### 2.2. Preparation of β-CD Functionalized Ag/Au-Rich Alloy NPs

Spherically shaped β-CD-Ag/Au-rich alloy NPs were prepared using an earlier reported method with slight modification [30,31]. The shape and size of the NPs were chosen to be spherical and around 25 nm for NP plasmonic resonance in the visible region. A schematic illustration of the synthetic protocol of the β-CD-Ag/Au-rich alloy NPs is given in Scheme S1. Briefly, 100 mL of AgNO_3_ (10 mM) was assorted with β-CD (0.185 g) containing 24.3 mL of doubly distilled water. To bring the pH of the solution to around ~10–12, 250 mL of NaOH (1.0 M) was injected after 5 min. Next, this reaction combination was dynamically stimulated on a heating stirrer, awaiting its color change from colorless to yellow, suggesting the creation of β-CD-AgNPs. To formulate β-CD-Ag/Au-rich alloy NPs, 250 mL of HAuCl_4_ (10 mM) was slowly injected into 25 mL of the β-CD-AgNPs solution with continuous stirring on heating. After about 30 min, the reaction mixture color altered from yellow to brownish-red, indicating that β-CD-Ag/Au-rich alloy NPs had formed. During the alloy NP construction reaction, the colloidal dispersion’s unique yellow color progressively weakened, whereas a brownish-red was established. To initiate the reduction reaction of Au^3+^ ions and their accumulation on the β-CD-AgNPs’ surface, neither extra NaOH nor β-CD was added. AgNPs were covered with Au^3+^ ions, owing to the basicity of the β-CD-AgNPs, and this helped the chemical reduction.

### 2.3. Sensitive and Specific Detection Protocol of CN^−^

For the spectrophotometric inspection of CN^−^ by the developed tactic, β-CD-Ag/Au-rich alloy NPs were used as such and directly. Next, the NaCN stock solution was newly made before utilization. In a 3 mL quartz cuvette, a notorious quantity of CN^−^ was introduced into 2.5 mL of β-CD-Ag/Au-rich alloy NPs. Then, these reaction mixtures were shaken well, and the reaction proceeded within 1 min. Therefore, without any delay, the CN^−^-added β-CD-Ag/Au-rich alloy NP solution was utilized to monitor for changes in the SPR band in the range from 800 to 350 nm. For the selectivity measurements, the same protocol was used, but different inorganic anions were supplied in place of CN^−^. Most importantly, photographs of the colorimetric variations of the β-CD-Ag/Au-rich alloy NPs with different inorganic species were taken at ambient conditions. Each spectrophotometric sensing and specificity measurement was conducted at least five times.

For the colorimetric assay-enabled RGB color variation assay of CN^−^, the reaction mixture (produced using the aforementioned process) was located with constant light and at a predetermined length (6 cm) from the mobile phone. The Realme 5 Pro phone, with a 48 MP back camera, was used as a detector. A color analysis program called Color Grap software version 3.9.2 was installed on the smartphone and utilized to measure the colorimetric variations in the β-CD-Ag/Au-rich alloy NPs’ colloidal dispersion in the introduction of increasing quantities of CN^−^. This program records RGB color variations using the camera and transforms them into values that are easy to understand. This technique was also performed five times to ensure correctness.

### 2.4. Real Sample Analysis

Under identical CN^−^ sensing settings, the real sample recovery test was conducted on drinking and tap water samples that had been spiked with CN^−^. The tap water was acquired in our laboratory directly, and the drinking water was purchased from a local market, Seongnam-si. For these water samples, pretreatment was not mandatory. As per the conventional addition protocol, a suitable section of each water sample was utilized for analysis. Lastly, the recovery studies were employed using three separate quantities of CN^−^ that were spiked with tap and drinking water samples. These cyanide-spiked water samples were mixed into the β-CD-Ag/Au-rich alloy NP colloidal dispersion, and the SPR band changes were monitored. Following that, the recovery test response against the samples of spiked water and the calibration curve (from the spectrophotometric method) was applied to calculate the CN^−^ concentration in these water samples. Consequently, the recovery values were calculated using the following formula [31,32]: Recovery (%) = (detected CN^−^/spiked CN^−^) × 100.

## 3. Results and Discussion

### 3.1. Structural Characterization of β-CD-Ag/Au-Rich Alloy NPs

**Figure 1:**
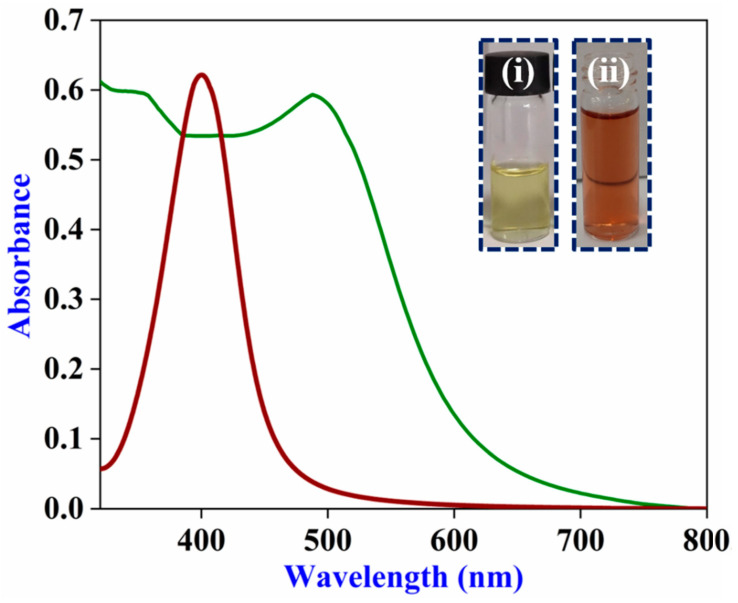
Absorbance spectra of β-CD-AgNPs and β-CD-Ag/Au-rich alloy NPs. The inset presents colorimetric photographs (**i**) and (**ii**) of β-CD-AgNPs and β-CD-Ag/Au-rich alloy NPs, respectively.

As stated in the experimental protocol, β-CD-Ag/Au-rich alloy NPs were synthesized by the wet chemical approach. The SPR bands are known to be a feature of both monometallic and bimetallic alloy nanoparticles [31,32]. Using a UV–vis absorption spectrum analysis, the SPR band features of the β-CD-Ag/Au-rich alloy NPs were examined to describe the optical features of NPs. Generally, core–shell nanoparticles (NPs) exhibit a double SPR band, whereas bimetallic alloy nanoparticles (BMNPs) often display a single SPR band [33,34]. First, the achieved yellow-color colloidal dispersion showed a unique SPR band at 402 nm as well as being yellow in color, which indicated the formation of β-CD-AgNPs (Figure 1 and inset Figure 1i). Furthermore, when the Au precursor content in the β-CD-AgNPs increased during the preparation of the β-CD-Ag/Au-rich alloy NPs, the SPR from the AgNPs side shifted spectrally towards the longer wavelength area. The SPR peak at 488 nm for the β-CD-Ag/Au-rich alloy NPs presented in Figure 1 fitted nicely with the composition of the β-CD-Ag/Au-rich alloy NPs. The accompanying images in the inset of Figure 1ii illustrated a progressive color shift from yellow to brownish-red as the Au content rose. These results validated the development of monodispersed β-CD-Ag/Au-rich alloy NPs in the present investigation. It was challenging to estimate the absolute shell thickness because of the near lattice match between the two metals (Ag and Au), and the relative shell thickness depended on the amount of HAuCl_4_ utilized [34,35]. This was because an alloy structure could easily form at the interface between the Ag and Au metals. The β-CD-Ag/Au-rich alloy NPs endured stability for three months when stored at room temperature without experiencing any color changes or changes to the solution’s SPR band (Figure S1). It was anticipated that the Au coating would increase the stability of BMNPs because Au acts as a barrier to oxidation of the Ag core [34,35,36].

**Figure 2:**
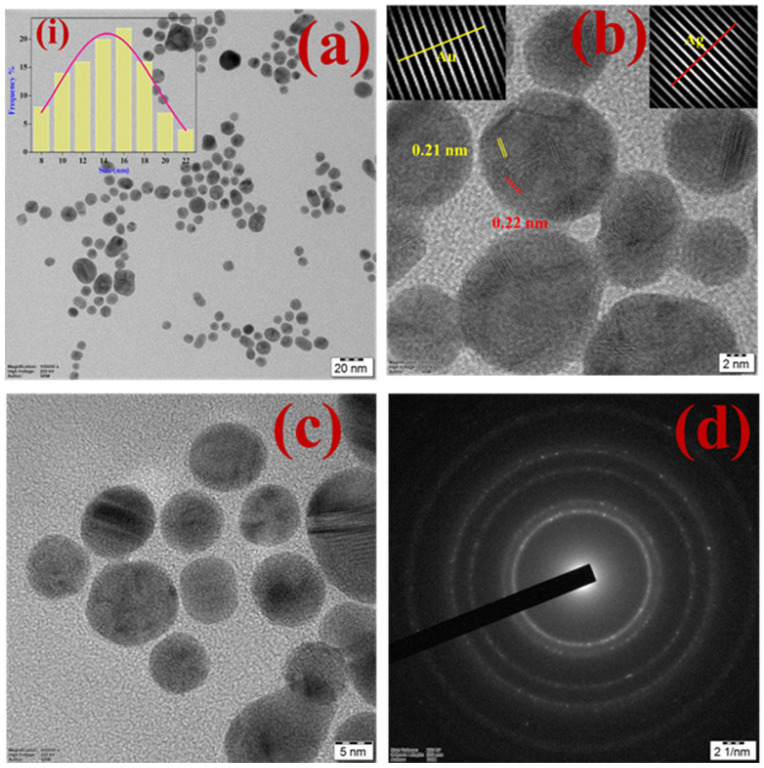
HR-TEM micrographs at different magnifications (**a**–**c**) and SAED pattern (**d**) of β-CD-Ag/Au-rich alloy NPs. Insets of (**a**,**b**) show the statistical analysis bar graph and lattice fringes of β-CD-Ag/Au-rich alloy NPs.

Using HR-TEM micrographs with different magnifications, the dimensions and morphologies of the attained β-CD-Ag/Au-rich alloy NPs were carefully examined, as displayed in Figure 2a–c. It was noted that there were many uniformly shaped, evenly distributed β-CD-Ag/Au-rich alloy NPs in the average size of about 16 ± 4 nm range (inset of Figure 2a). Furthermore, HR-TEM images of the β-CD-Ag/Au-rich alloy NPs demonstrated that the two different lattice fringes, (Au = 0.21 nm) and (Ag = 0.22 nm), indicated the crystalline nature and the presence of Au/Ag elements in the composition of BMNPs (inset of Figure 2b and Figure S2a,b). A selective area energy dispersion (SAED) pattern was used to study the structural makeup of β-CD-Ag/Au-rich alloy NPs (Figure 2d). Debye–Scherrer rings are well-defined in the SAED patterns of β-CD-Ag/Au-rich alloy NPs, designating the NPs as crystalline in nature [37]. The β-CD-Ag/Au-rich alloy NPs’ bimetallic alloy structure was addressed using the energy dispersive X-ray (EDX) spectral approach. The compositional data of the β-CD-Ag/Au-rich alloy NPs exhibited the presence of Au (89.61%) and Ag (10.39%) elements (Table S1) in the EDX spectrum, which indicated that the bimetallic nanoparticles were made up of Au and Ag atoms (Figure S2).

Dynamic light scattering (DLS) and zeta potential measurements were employed to inspect the average hydrodynamic diameter and surface charge values of the β-CD-Ag/Au-rich alloy NPs. DLS analysis was applied to estimate the size of colloidal NPs because of their continual Brownian motion, which causes time-dependent variations in the scattering strength that DLS detects when concerning the particle size. The mean hydrodynamic diameter of 25 nM was displayed, signifying that β-CD-Ag/Au-rich alloy NPs are homogenous and uniform in size (with a NP diameter of about 25 nm on average) (Figure S3). The mean NP size estimated from the DLS and HR-TEM outcomes presented a noticeable discrepancy. This could be explained by the fact that HR-TEM measured the size of the NPs in a dried state, while DLS monitored the hydrodynamic radii of the NPs in a colloidal medium [37]. The zeta potential was utilized to inspect the durability of colloidal NPs concerning the charge on their surfaces. Generally, the zeta potential surface charge was bigger than +30 mV or lower than −30 mV for t demonstrated extremely stable colloidal NPs [38,39]. Figure S4 represents the zeta potential data of β-CD-Ag/Au-rich alloy NPs. As depicted in Figure S4, the β-CD-Ag/Au-rich alloy NPs exhibited a surface charge value of −39.21 mV, illustrating a high stability of β-CD-Ag/Au-rich alloy NPs in the dispersion state [40,41].

**Figure 3:**
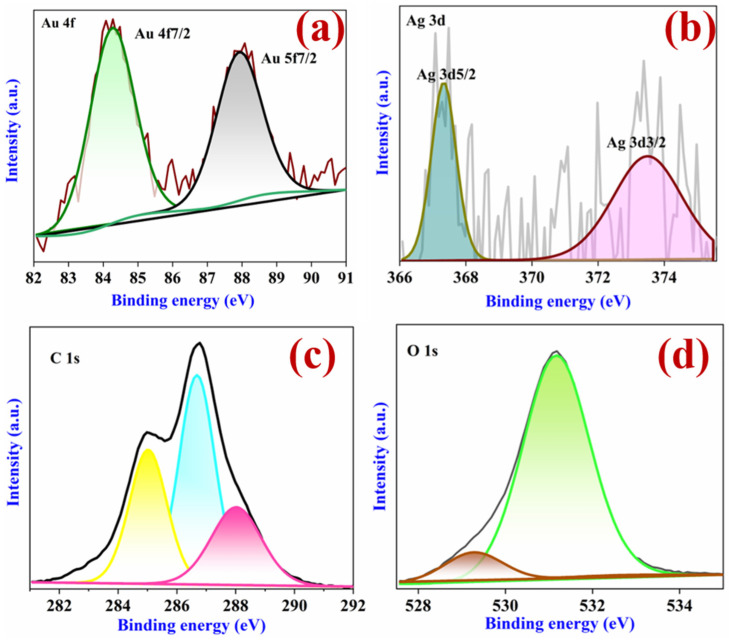
High-resolution XPS spectra of Au (**a**), Ag (**b**), C (**c**), and O (**d**) elements.

The surface alloy chemical states of the Au and Ag elements in β-CD-Ag/Au-rich alloy NPs were verified by XPS investigation, as given in Figure S5. As depicted in Figure S5, the XPS full scan survey spectrum of the β-CD-Ag/Au-rich alloy NPs displayed the occurrence of C (284.98 eV), O (531.02 eV), Ag (367.33 eV), and Au (84.26 eV) elements. Furthermore, high-resolution XPS spectra of the C 1s, O 1s, Au 4f, and Ag 3d elements are displayed in Figure 3. It has been observed that a doublet band in each case emerged at binding energies characteristic of the matching metal with an oxidation state of zero. In particular, deconvoluted XPS spectra of the Au 4f peak showed a doublet made up of the Au 4f7/2 and Au 4f5/2 peaks, which were positioned at the 84.25 and 87.93 eV binding energies, respectively (Figure 3a). Additionally, the dual peaks detected in the Ag 3d high-resolution XPS spectra at 367.34 and 373.49 eV binding energies could be attributed to the Ag 3d5/2 and Ag 3d3/2, respectively (Figure 3b). The binding energy gaps between the Au 4f7/2 and Au 4f5/2 peaks (Δ = 3.7 eV), along with the energy gap between the Ag 3d5/2 and Ag 3d3/2 peaks (Δ = 6.0 eV), were identical values for Au (0) and Ag (0). The observed binding energy values of the Au and Ag elements were good and in accordance with previously published works [42,43,44]. In the high-resolution spectrum of C 1s, peaks at 285.02, 286.67, and 287.97 eV were allocated to C−C, C−O−C, and O−C = O, respectively (Figure 3c). In the O 1s deconvoluted XPS spectra, C−OH and Ag−O were identified as the sources of the peaks at 531.15 and 529.07 eV, respectively (Figure 3d). These outcomes further validated that β-CD effectively binds on the surface of Ag/Au-rich alloy NPs [42].

### 3.2. Optimization of the Present Work

Many aspects, such as the effect of the solution’s pH and the assay’s reaction time, were investigated and optimized to yield the best sensing efficiency. Firstly, the β-CD-Ag/Au-rich alloy NPs were highly stable in an alkaline medium with more than pH = 8, and they were unstable in an acidic medium with a lower than equal pH = 7. In addition, in an acidic or neutral medium, CN^−^ could also be converted into a volatile HCN species. As shown in Figure S6, at a pH ranging from 8 to 11, the colorimetric assay showed good sensitivity for CN^−^. As a result, we selected pH 9 as the ideal value for the current sensing strategy. The assay’s response time was also examined, revealing (Figure S7) that the reaction between CN^−^ and β-CD-Ag/Au-rich alloy NPs could be completed in less than one minute. Consequently, the current sensing tactic’s ideal reaction time was approximately one minute. Sodium chloride (NaCl) was used to examine the impact of ionic strength on the detecting system. The β-CD-Ag/Au-rich alloy NPs remained stable as the NaCl concentration rose from 0 to 100 mM, as noted in Figure S8. This result implied that NaCl had a negligible impact on the CN^−^ sensing assay’s performance. The amount of β-CD-Ag/Au-rich alloy NPs was optimized to be 2.5 mL for the best sensitivity in detecting CN^−^ while maintaining sensing stability.

### 3.3. Spectrophotometric and Colorimetric Detection of CN^−^

**Figure 4:**
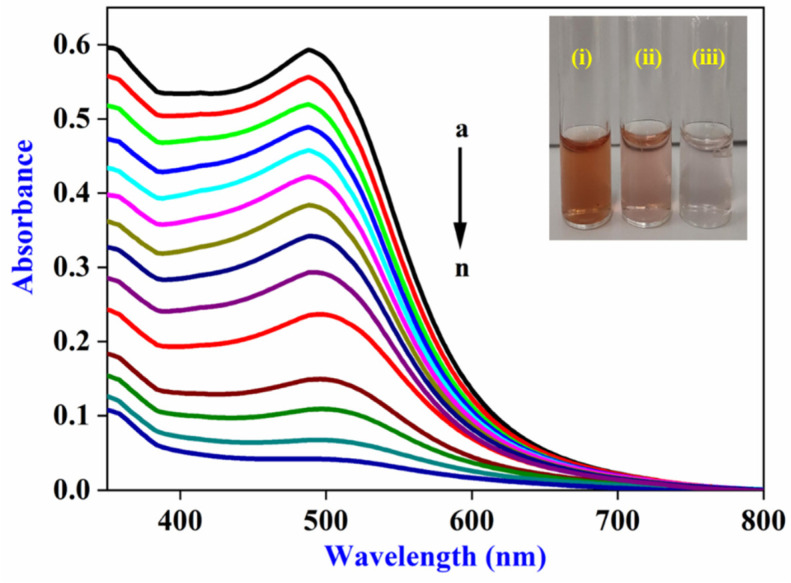
SPR band alteration of β-CD-Ag/Au-rich alloy NPs with incremental quantities of CN^−^. [CN^−^] = 7.5–97.5 nM; each addition, 7.5 nM. The inset displays colorimetric photographs of β-CD-Ag/Au-rich alloy NPs without (**i**) and with different quantities of 45 (**ii**) and 90 nM (**iii**) CN^−^.

The primary goal of creating β-CD-Ag/Au-rich alloy NPs was to employ them in high-sensitivity CN^−^ quantification. Using the plasmonic and colorimetric features, the impact of CN^−^ on β-CD-Ag/Au-rich alloy NPs in an aqueous medium was examined, and the typical equilibrium process was displayed in Figure 4. Before introducing CN^−^, the β-CD-Ag/Au-rich alloy NPs exhibited a distinguishing LSPR band at 488 nm and a strong brownish-red color. Upon injecting increasing quantities of CN^−^ solutions to the β-CD-Ag/Au-rich alloy NPs’ colloidal dispersion, the SPR band intensities reduced, with a red shift from 488 nm to 496 nm noticed. At the same time, there was a conspicuous color difference, from a strong brownish-red to light brownish-red (45 nM) to colorless (90 nM), which was easily checkable by the naked eye (inset of Figure 4). The minimal CN^−^ concentration that was visually distinguishable was 45 nM, while the maximum allowed cyanide amount in drinking water, set by the WHO, was 1.9 mM. In addition, the colorimetric approach performed better in detecting CN− in drinking water. The notable variations in the spectrum and the colorimetric measurement indicated a strong etching reaction that was involved between the β-CD-Ag/Au-rich alloy NPs and CN^−^. Based on the suppression of the SPR band, the quantities of CN^−^ could be computed. Furthermore, absorption spectrophotometry and naked-eye inspection were adequate for performing an analysis of the visual-based sensing tactics without the need for complex equipment. It was noted that the absorbance spectrum was not subject to incident polarization of light interacting with NPs due to the random nature of the orientation of NPs in a colloid despite the presence of unexpected anisotropy of particle shapes.

### 3.4. Sensing Mechanism

The following two chemical reactions served as the foundation for the SPR band and colorimetric fluctuations for β-CD-Ag/Au-rich alloy NPs with CN^−^ [22,26].

4Au + 8CN^−^ + 4H_2_O + O_2_ = 4[Au(CN)_2_]^−^ + 4OH^−^

4Ag + 8CN^−^ + 4H_2_O + O_2_ = 4[Ag(CN)_2_]^−^ + 4OH^−^

**Scheme 1:**
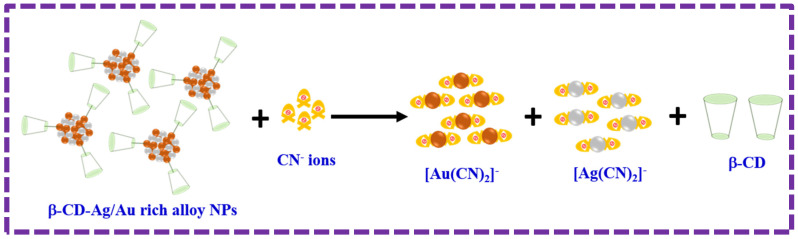
Schematic illustration of the sensing mechanism for the detection of CN^−^ by β-CD-Ag/Au-rich alloy NPs.

**Figure 5:**
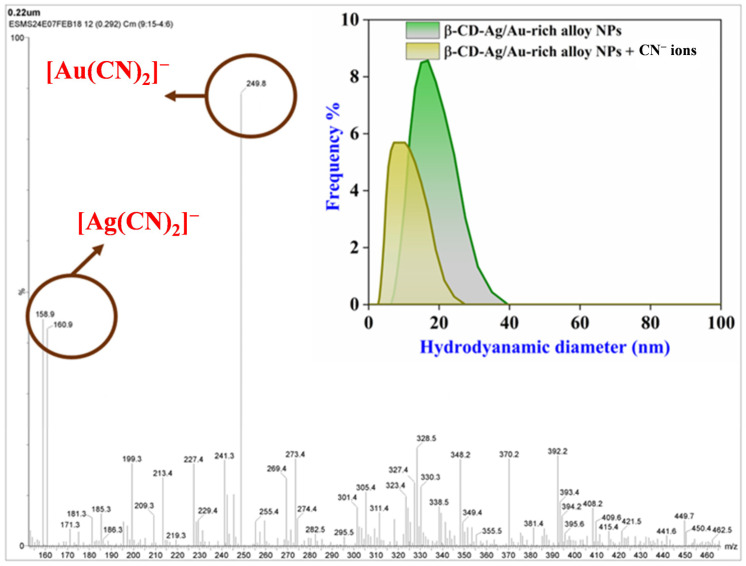
ESI-mass spectral outcomes of β-CD-Ag/Au-rich alloy NPs with 75 nM of CN^−^ and the inset shows the DLS investigation results for β-CD-Ag/Au-rich alloy NPs with 75 nM of CN^−^.

In the presence of oxygen, CN^−^ was expected to successfully etch both metal Ag and Au, changing the alloy composition ratio and causing detectable spectrum variations as well as changes in visual color, as seen in Scheme 1. Accordingly, we injected CN^−^ into the β-CD-Ag/Au-rich alloy NP colloidal dispersion. The reaction mixture quickly turned from brownish-red to colorless, as expected. Concurrently, there was a red shift in the SPR band from 488 to 496 nm, indicating the destruction of the β-CD-Ag/Au-rich alloy NPs. The HR-TEM measurement further confirmed this proposed mechanism. After adding CN^−^, it was discovered that the β-CD-Ag/Au-rich alloy NPs were etched, as seen in the TEM micrographs (Figure S9a,b). The DLS measurement findings illustrated that the average hydrodynamic diameter reduced from 25 nm to 7 nm, also confirming CN^−^ etching on the surface of β-CD-Ag/Au-rich alloy NPs (Inset of Figure 5). In addition, the products of the reaction between β-CD-Ag/Au-rich alloy NPs and CN^−^ were identified by ESI-mass spectroscopy (before the addition of CN^−^, the corresponding peaks related to the [Au(CN)_2_]^−^/[Ag(CN)_2_]^−^ were not identified). β-CD-Ag/Au-rich alloy NPs, in the presence of 75 nM CN^−^—a fragment peak at *m*/*z* = 249.8 associated with [Au(CN)_2_]^−^—was produced, and two additional distinct peaks associated with it were found at *m*/*z* = 160.9 and 158.9. This signifies the CN^−^ induced decomposition of β-CD-Ag/Au-rich alloy NPs (Figure 5) [26]. These findings indicated that after the introduction of CN^−^ into β-CD-Ag/Au-rich alloy NPs, CN^−^ destroyed the morphological and optical features of the β-CD-Ag/Au-rich alloy NPs via metal-cyano complex formation.

### 3.5. Selectivity

**Figure 6:**
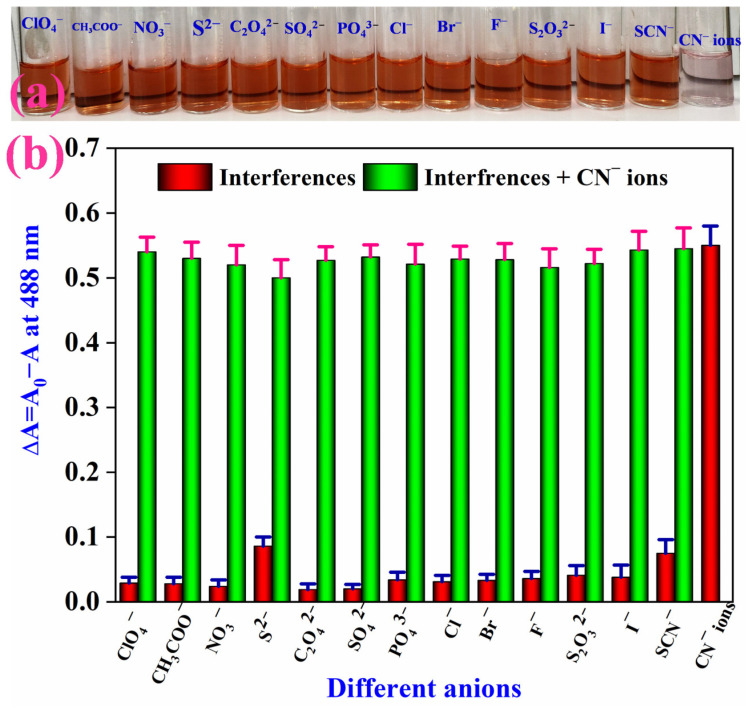
The visual color changes of the colorimetric response (**a**) and optical absorbance changes (**b**) for β-CD-Ag/Au-rich alloy NPs with different anions. The red bar represents β-CD-Ag/Au-rich alloy NPs with various anions, while the green bar represents β-CD-Ag/Au-rich alloy NPs with different anions plus CN^−^.

The CN^−^ detection specificity with the β-CD-Ag/Au-rich alloy NPs was examined using co-existing interfering anions (Br^−^, ClO_4_^−^, NO_3_^−^, S^2−^, C_2_O_4_^2−^, SO_4_^2−^, PO_4_^3−^, Cl^−^, CH_3_COO^−^, F^−^, S_2_O_3_^2−^, I^−^, and SCN^−^) under identical experimental conditions. The concentration of CN^−^ was 100 nM, and those of other interfering substances were 10 times (1 mM) that of CN^−^, whereas the amounts of S^2−^and SCN^−^ were 20 times (2 mM) that of CN^−^. Interfering molecules induced the SPR fluctuations (bar graph), and the colorimetric variations are displayed in Figure 6. Except for CN^−^, other potential interfering species did not alter the colorimetric features of the β-CD-Ag/Au-rich alloy NPs, even at concentrations that were 10- or 20-fold higher than CN^−^ (Figure 6a). The relative SPR band intensity (DA = A_0_-A, A_0_, and A denotes the SPR band intensity measured before and after adding the analyte) in the β-CD-Ag/Au-rich alloy NPs with CN^−^ were notably high, but other potential interfering species had no noticeable change on the SPR band of the β-CD-Ag/Au-rich alloy NPs, even at concentrations that were 10- or 20-fold higher than that of CN^−^. Additionally, competitive experiments were conducted to further authenticate the specificity performance of the plasmonic nanoprobe, in which CN^−^ and other interfering species were mixed into the β-CD-Ag/Au-rich alloy NPs colloid. After inserting CN^−^ into the solution of β-CD-Ag/Au-rich alloy NPs + other interferents, the SPR band apparently reduced, which was nearly equal to the case of injecting CN^−^ alone. This indicated that the present approach was capable of distinguishing CN^−^ from other potential anionic species. Such high specificity for CN^−^ could be due to the equilibrium constant for the chemical interaction between Au and CN^−^ (4.4 × 10^28^) and Ag and CN^−^ (9.4 × 10^24^). Therefore, the dissolution of β-CD-Ag/Au-rich alloy NPs could arise from a significantly lower dosage of CN^−^ that etched the surface of β-CD-Ag/Au-rich alloy NPs.

### 3.6. Smartphone-Based Colorimetry

**Figure 7:**
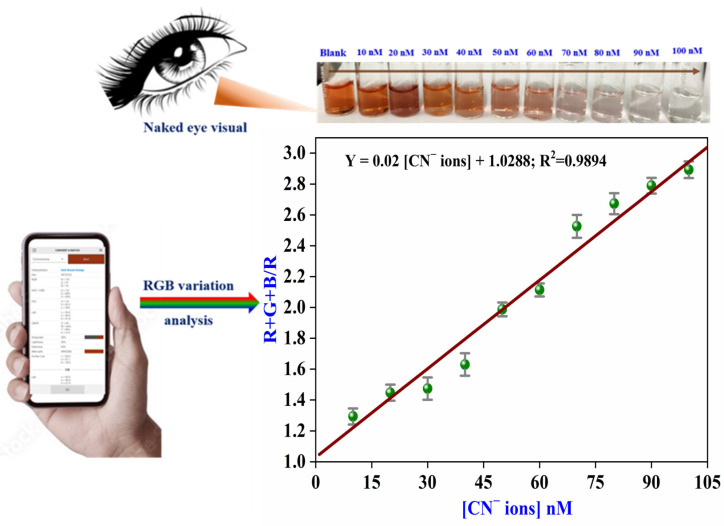
The ratio of the sum of the smartphone R, G, and B values to R values as a function of CN^−^. These RGB values were taken from the colloids of β-CD-Ag/Au-rich alloy NPs in the presence of CN^−^ using cameras and the RGB software of a smartphone. CN^−^ concentration ranges from 10 to 100 nM; each addition is 10 nM.

The use of smartphones allows for significant advantages over traditional approaches, including cost-effectiveness, ease of use, speedy analysis, and compact design, in addition to portability and real-time monitoring purposes [45,46,47]. The presented technologies of plasmon-induced colorimetry were combined with a smartphone where cameras and color sensors were available. The gradual visual color change in the colloids of the β-CD-Ag/Au-rich alloy NPs with an increasing concentration of CN^−^ took place, from brownish-red to colorless. The smartphone-based colorimetric method relied on the recognition of RGB (red-blue-green) values that ranged from 0 to 255 for each color. The values [255,255,255] and [0,0,0] on this RGB scale stood for complete white and black, respectively [48,49]. The photographs of the colloids of the β-CD-Ag/Au-rich alloy NPs were taken with an increasing CN^−^ concentration by the back camera. These photos were then analyzed with a Color Grap application loaded onto the smartphone. This application produced the RGB values as the gradual color changed upon the injection of CN^−^ into β-CD-Ag/Au-rich alloy NPs (Table S2). We used the arithmetic ratio between the sum of the R, G, and B values and the R value to display as a function of the CN^−^ concentration, as shown in Figure 7. Linear regression was also obtained in the concentrations ranging from 10 to 100 nM, with R^2^ = 0.9894. Using smartphone-assisted RGB techniques, the LOD of CN^−^ was estimated as 1.35 nM. The LOD turned out to be much less than the WHO-set maximum threshold of CN^−^ in drinking water. It was seen that this technique, based on a smartphone, lent itself to the cost-effective monitoring of CN^−^ present in a water source in a real-time and in situ manner without compromising sensitivity and successfully detected CN^−^ both quantitatively and visually.

### 3.7. Analytical Parameters

**Figure 8:**
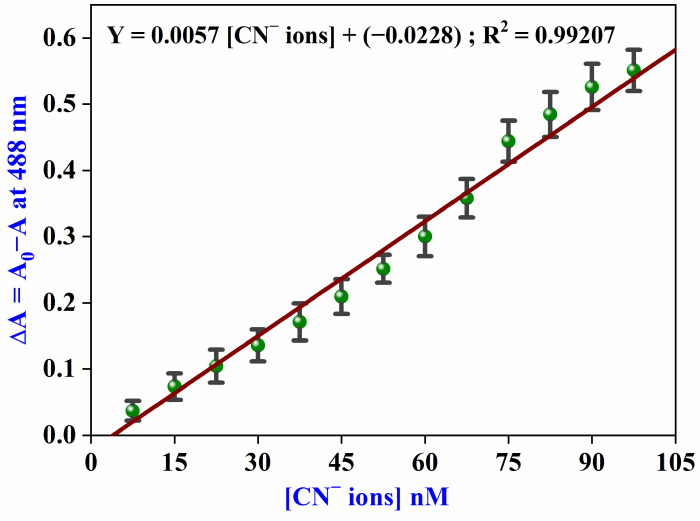
Calibration plot for β-CD-Ag/Au-rich alloy NP-based spectrophotometric detection of CN^−^.

Figure 8 shows the optical absorbance change as a function of the CN^−^ concentration with its linear fit (solid curve) for the colloids of β-CD-Ag/Au-rich alloy NPs. The absorbance change was measured by comparing the absorbance between before and after injections of CN^−^ with a given concentration using a spectrophotometer. Linear regression was obtained to fit the data in the range from 7.5–97.5 nM concentrations with an R^2^ of 0.99207 (Figure 8). This led us to estimate the LOD of CN^−^ as 0.24 nM at a signal-to-noise ratio of 3, being remarkably smaller than the acceptable level of CN^−^ (1.90 mM) in drinkable water set by the WHO. This indicated that the β-CD-Ag/Au-rich alloy NP-based CN^−^ sensor with a spectrophotometer provided a sensitivity higher than that of a smartphone.

Table S3 shows a comparison between the characteristics of CN^−^ detection using various approaches with Au/Ag bimetallic nanostructures, including the current work. While the previously reported ones in Table S3 had a Ag@Au core–shell structure, our Ag/Au bimetallic nanoprobes were the alloy that could modify the plasmonic properties in a different manner. It turned out that, when using a spectrophotometer for sensing, the Ag/Au alloy NPs produced a LOD that was two or three orders of magnitude lower than those of the previously reported Ag/Au NPs used in colorimetric approaches [5,22,26,50,51]. This indicated that the current nanoprobes of the Ag/Au alloy NPs outperformed the Ag/Au core–shell nanoprobes in terms of sensitivity when employing colorimetric approaches. It was also revealed that, although the smartphone-based approach yielded a LOD that was one or two orders of magnitudes higher than those of the other approaches in Table S3, it benefitted the sensing platform, in that it could be potentially fabricated as extremely light, cost-effective, as well as being a tiny portable device with no compromise of sufficient sensitivity [32,52].

### 3.8. Practical Utility and Reproducibility

For the systematic efficacy of the current spectrophotometric method in sensing CN^−^ in environmental water samples, we selected tap and drinking water samples for this test. Although the targeted CN^−^ was initially absent from the chosen water samples, we have confirmed the environmental water samples through spiked recovery studies. Next, tap and drinking water samples were spiked with three different notorious quantities of CN^−^. CN^−^-spiked water samples were applied to β-CD-Ag/Au-rich alloy NPs, and absorbance spectral studies/RGB color variation analyses were carried out. Finally, Table S4 provides a summary of the findings from these analyses as well as the recoveries for the tampered samples. From Table S4, it is evident that the spectrophotometric system’s recovery ranged below 93.1% and 99.1% of its maximum with an RSD of <2.98%, and the RGB color variation investigation provided a recovery from 93.8% to 98.8% with an RSD of <3.07%. The measured and spiked CN^−^ levels closely matched the observed findings. The results of this smartphone-enabled spectrophotometric method indicate that β-CD-Ag/Au-rich alloy NPs show promise for the use of CN^−^ detection in natural water samples. Furthermore, we checked the reproducibility of CN^−^ sensing with 3-month-aged alloy NPs using a spectrophotometer. It was found that no meaningful change was observed when compared to freshly arranged ones (see Figure S1), supporting that the present sensing technique benefitted from the stability and reproducibility of the sensing properties over time.

## 4. Conclusions

Colorimetric assays of CN^−^ in water specimens were demonstrated using hybrid plasmonic nanoprobes, i.e., β-CD-Ag/Au-rich alloy NPs with spectrophotometric instruments and a smartphone device. Excellent performance, both in sensitivity and great specificity, was observed, with LODs of 0.24 nM (spectrophotometry) and 1.35 nM (smartphone RGB color assay), in a very straightforward way compared to the conventional HPLC-based assay. Spherically/quasispherically shaped β-CD-Ag/Au-rich alloy NPs with good dispersion were successfully prepared, as verified by characterization methods such as absorbance, FT-IR, XPS, DLS, HR-TEM, and zeta potential measurements. These alloy NP visual probes, upon interaction with CN^−^, exhibited notable suppression of the plasmonic band, manifesting a color shift from brownish-red to colorless. This could be attributed to the chemical response between the β-CD-Ag/Au-rich alloy NPs and CN^−^, producing metal-cyano complexes, i.e., [Au(CN)_2_]^−^/[Ag(CN)_2_]⁻, and demolishing the β-CD-Ag/Au-rich alloy NPs morphology. This allows for the selective identification of CN^−^ across a broad concentration range from 7.5 to 97.5 nM, with a linear fitting coefficient of 0.99207 and a LOD of 0.24 nM, using a spectrophotometer. The color change accompanied by the plasmonic band variations enabled a smartphone capability of color sensing to be exploited for its potential application for on-site real-time monitoring of CN^−^. This portable scheme allowed a wide linear range of detection, from 10 to 100 nM with an LOD of 1.35 nM, which is still lower than the minimum set by the WHO (1.91 nM) for drinking water. The present technique of using a smartphone device holds great promise for monitoring CN^−^ simplistically and cost-effectively and can be used as an effective means for escalating the preservation of safe water sources present in ecological systems.

Together with artificial intelligence technologies, the presented technologies could play a pivotal role in improving global water safety, environmental health monitoring, and industrial waste management [53]. This can provide a cost-effective and simple-to-use system for the on-site detection of CN^−^ in water sources, an important step toward tackling cyanide contamination on a global scale.

## Data Availability

The original contributions presented in this study are included in the article/Supplementary Material. Further inquiries can be directed to the corresponding authors.

## Supplementary Materials

The following supporting information can be downloaded at: https://www.mdpi.com/article/10.3390/ma18071604/s1, and this file contains a schematic illustration of synthesis of NPs (Scheme S1: Schematic representation of synthetic protocol of β-CD-Ag/Au-rich alloy NPs), stability of alloy NPs over time (Figure S1: Absorbance spectra of freshly and 3-months aged β-CD-Ag/Au-rich alloy NPs,with an insent showing the corresponding colorimetric photographs), Lattice fringes and EDX spectrum of alloy NPs (Figure S2: Lattice fringes calculated values by ImageJ software results and EDX spectrum of β-CD-Ag/Au-rich alloy NPs), DLS results for alloy NPs (Figure S3: DLS data for β-CD-Ag/Au-rich alloy NPs), Zeta potential results for alloy NPs (Figure S4: Zeta potential result for β-CD-Ag/Au-rich alloy NPs), XPS survey spectrum (Figure S5: XPS survey spectrum of β-CD-Ag/Au-rich alloy NPs), effect of pH on sensing (Figure S6: Effect of pH on β-CD-Ag/Au-rich alloy NPs with 7.5 nM of CN^−^), response time data (Figure S7: Effect of response time on β-CD-Ag/Au-rich alloy NPs with 7.5 nM of CN^−^), SPR band change by NaCl (Figure S8: SPR band changes of β-CD-Ag/Au-rich alloy NPs with incremental amounts of NaCl). Figure S9: HR-TEM micrographs of βCD-Ag/Au-rich alloy NPs with 60 nM of CN^−^ at different magnifications. Additionally, this file contains EDX data (Table S1: EDX data for β-CD-Ag/Au-rich alloy NPs), RGB color values obtained from smartphones (Table S2: RGB color variation results for β-CD-Ag/Au-rich alloy NPs with different quantities of CN^−^ ions), a comparison table of the present work with previously reported methods (Table S3: Comparison between the analytical parameters of the present work with previously reported Au/Ag bimetallic nanostructures), and real water analysis findings (Table S4: Real water sample results for CN^−^ ions quantification based on β-CD-Ag/Au-rich alloy NPs by the SPR-based colorimetric platform and smartphone-based RGB color values tactics).
