# Supplementary material for: Smartphone-Assisted Plasmonic Nanosensor for Visual and Specific Sensing of Toxic Cyanide Ions by β−Cyclodextrin Templated Gold-Rich/Silver Bimetallic Alloy Nanoparticles
Source: Materials (Basel). 2025 Apr 2;18(7):1604. doi: 10.3390/ma18071604 (PMC11990465; doi:10.3390/ma18071604)
Supplement: Supplementary file 1 [file materials-18-01604-s001.zip › materials-3535897-supplementary.pdf]

Electronic supporting information

Smartphone-assisted plasmonic nanosensor for visual and specific sensing of toxic cyanide ions by β–Cyclodextrin templated gold-rich/silver bimetallic alloy nanoparticles

Nguyen Nam Phuong Truong[1,2], Ramar Rajamanikandan[1,2]*, Kandasamy Sasikumar[1,2], Heongkyu Ju[1,2]**

Department of Physics and Semiconductor Science, Gachon University, Seongnam-si, Republic Korea, 13120.

Gachon Bionano Research Institute, Gachon University, Seongnam-si, Republic Korea, 13120.

* Co-corresponding author: chemistrmkd@gachon.ac.kr

** Corresponding author: batu@gachon.ac.kr

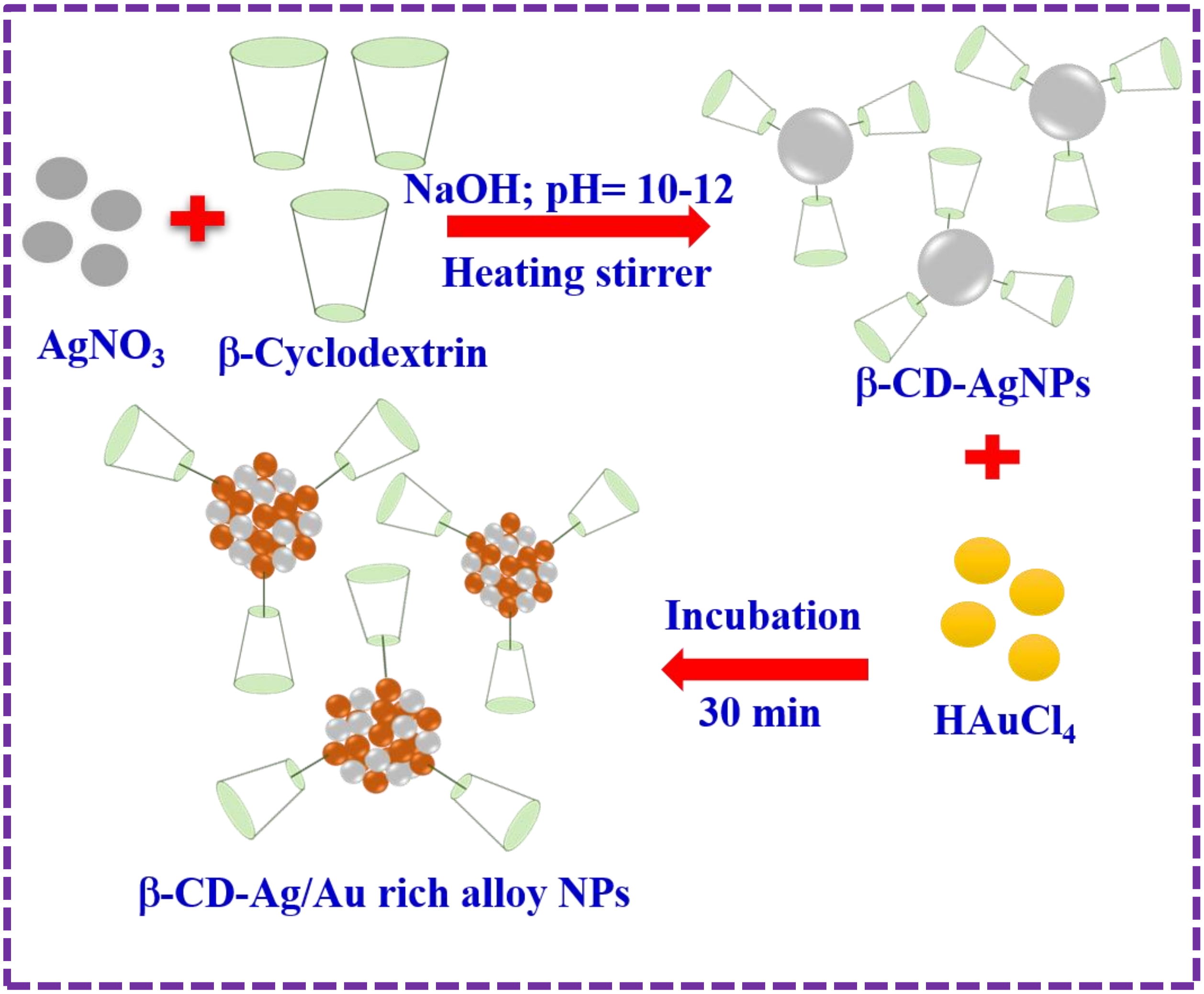

Scheme S1. Schematic representation of synthetic protocol of β–CD-Ag/Au-rich alloy NPs.

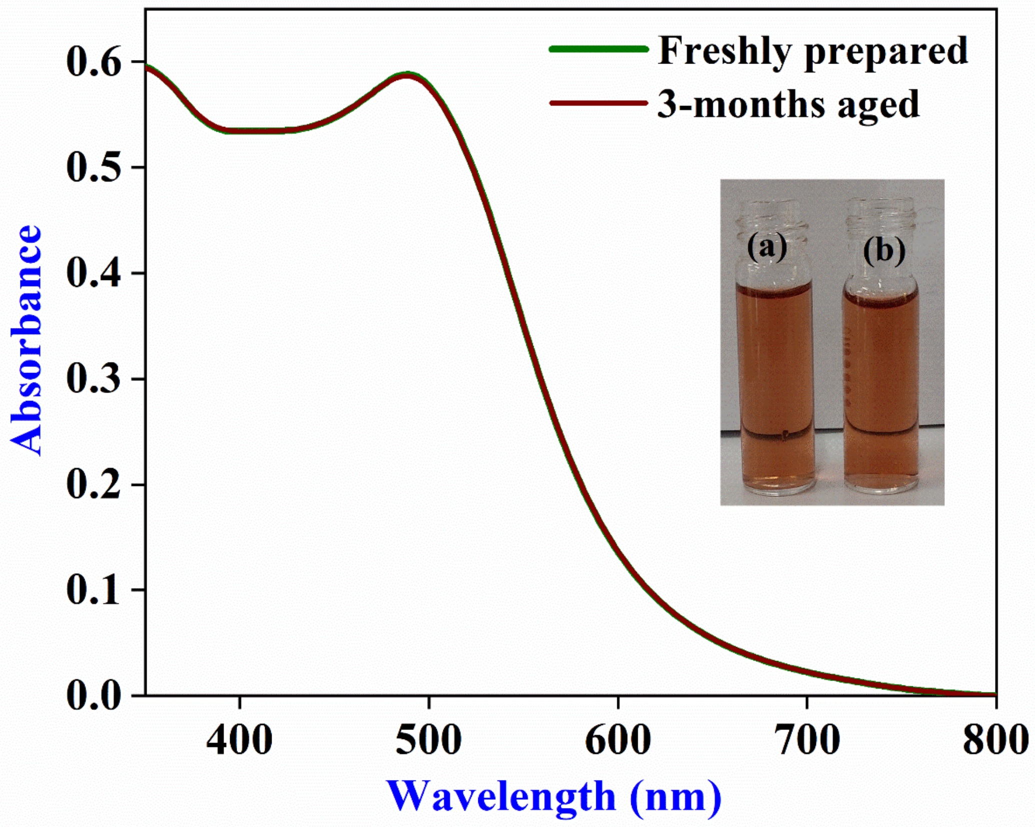

Figure S1. Absorbance spectra of freshly and 3-months aged β−CD-Ag/Au-rich alloy NPs and inset show corresponding colorimetric photographs.

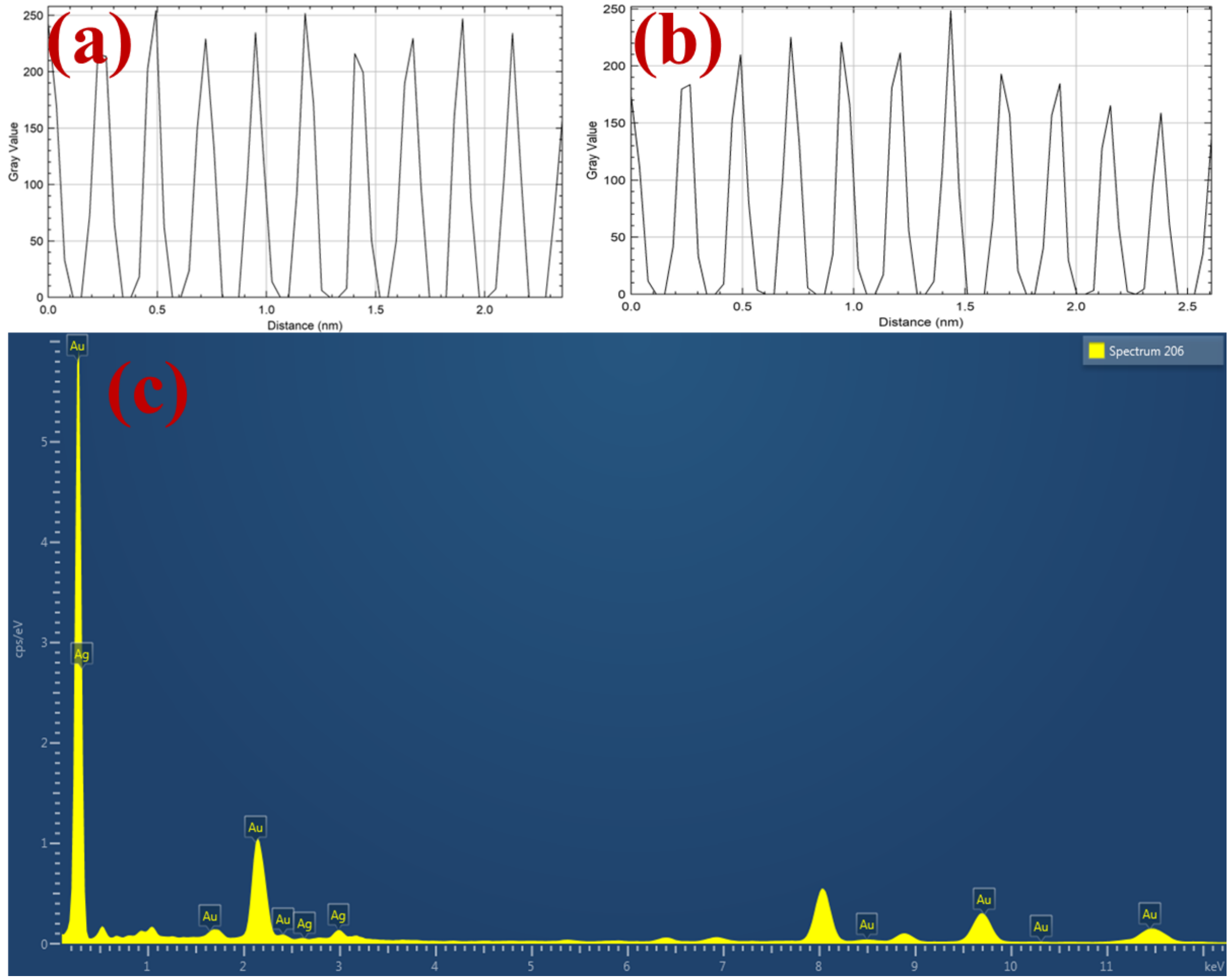

Figure S2. Lattice fringes calculated values by ImageJ software results and EDX spectrum of β–CD-Ag/Au-rich alloy NPs

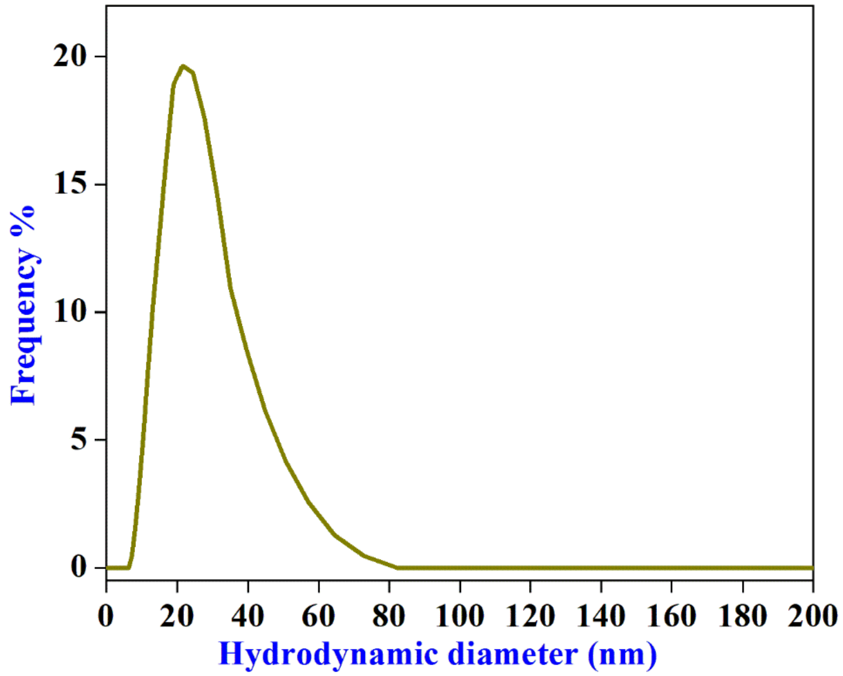

Figure S3. DLS data for β–CD-Ag/Au-rich alloy NPs.

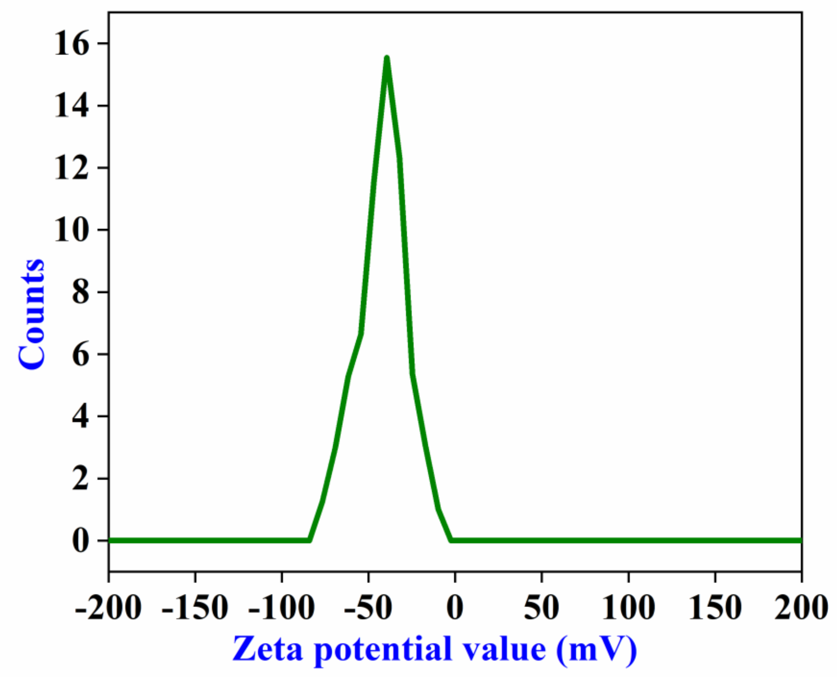

Figure S4. Zeta potential result for β−CD-Ag/Au-rich alloy NPs

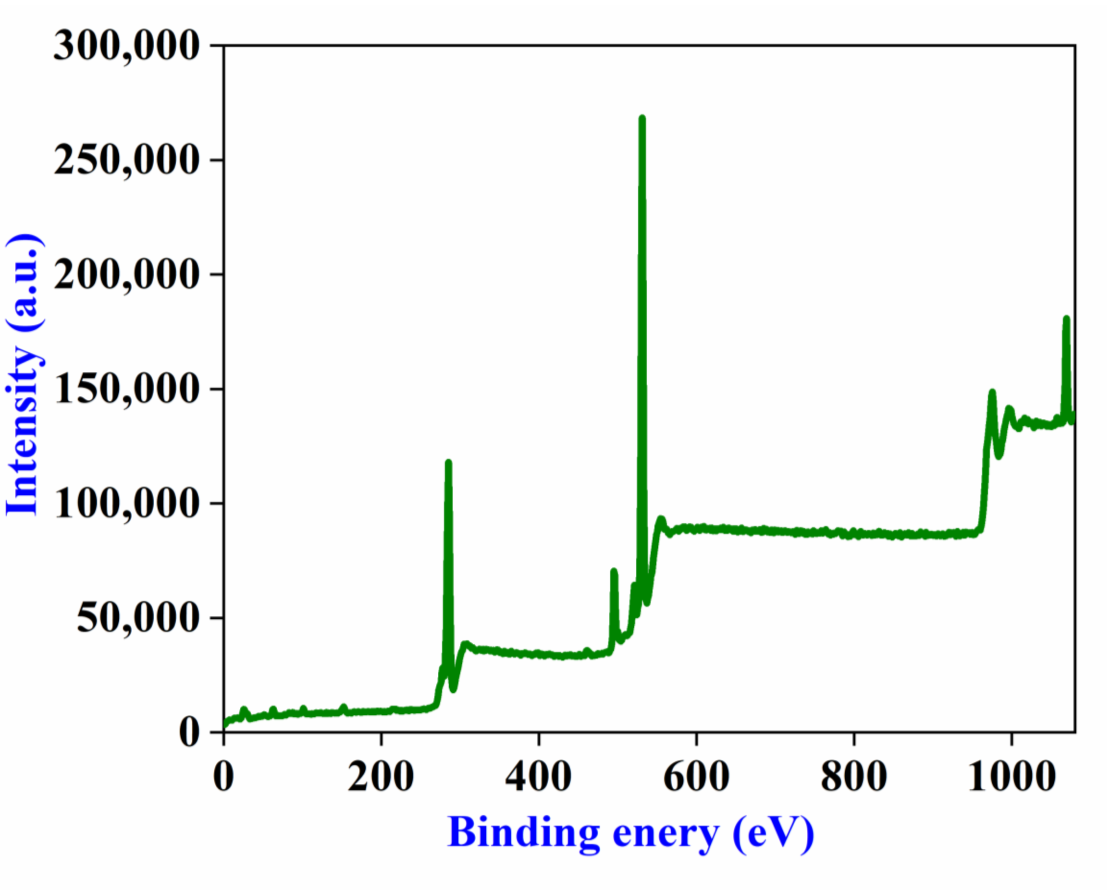

Figure S5. XPS survey spectrum of β−CD-Ag/Au-rich alloy NPs.

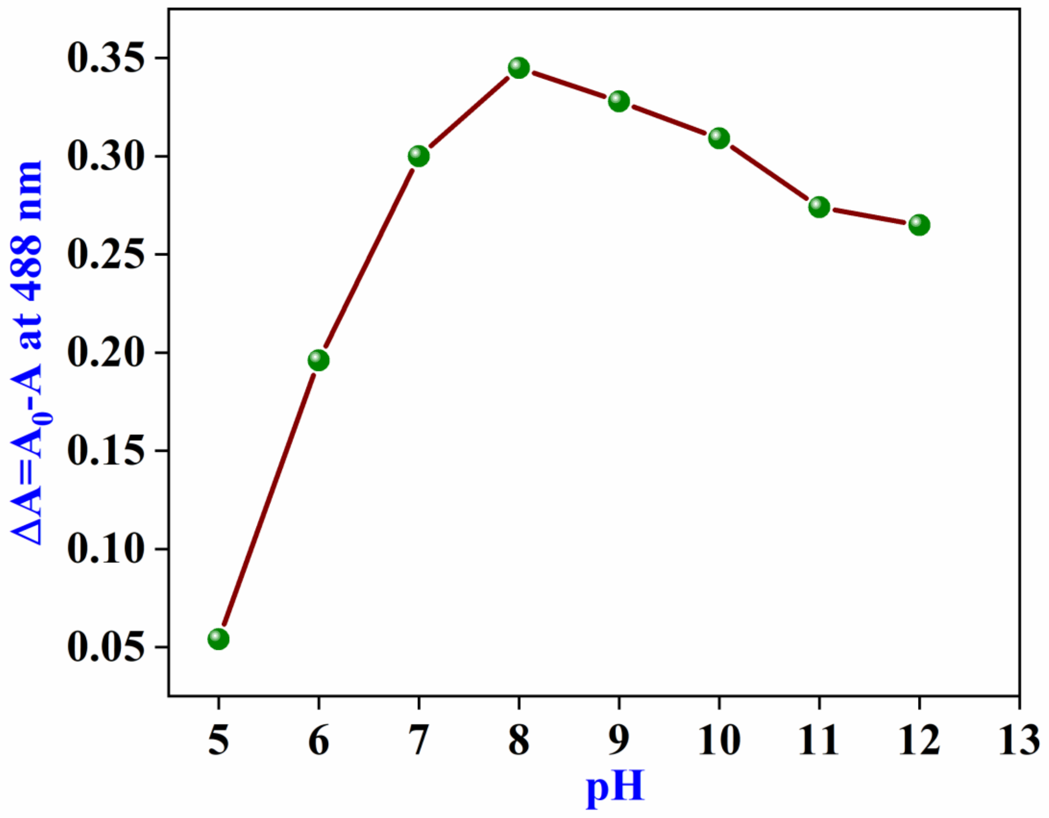

Figure S6. Effect of pH on β–CD-Ag/Au-rich alloy NPs with 7.5 nM of CN^{-}.

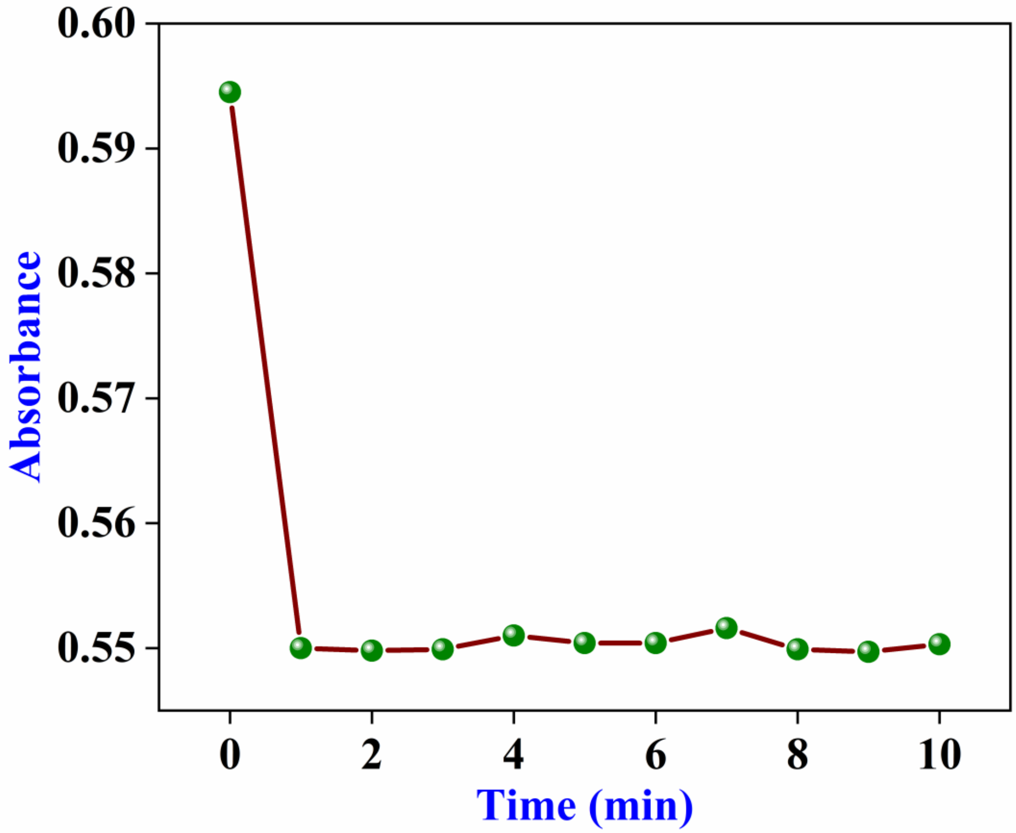

Figure S7. Effect of response time on β−CD-Ag/Au-rich alloy NPs with 7.5 nM of CN^-.

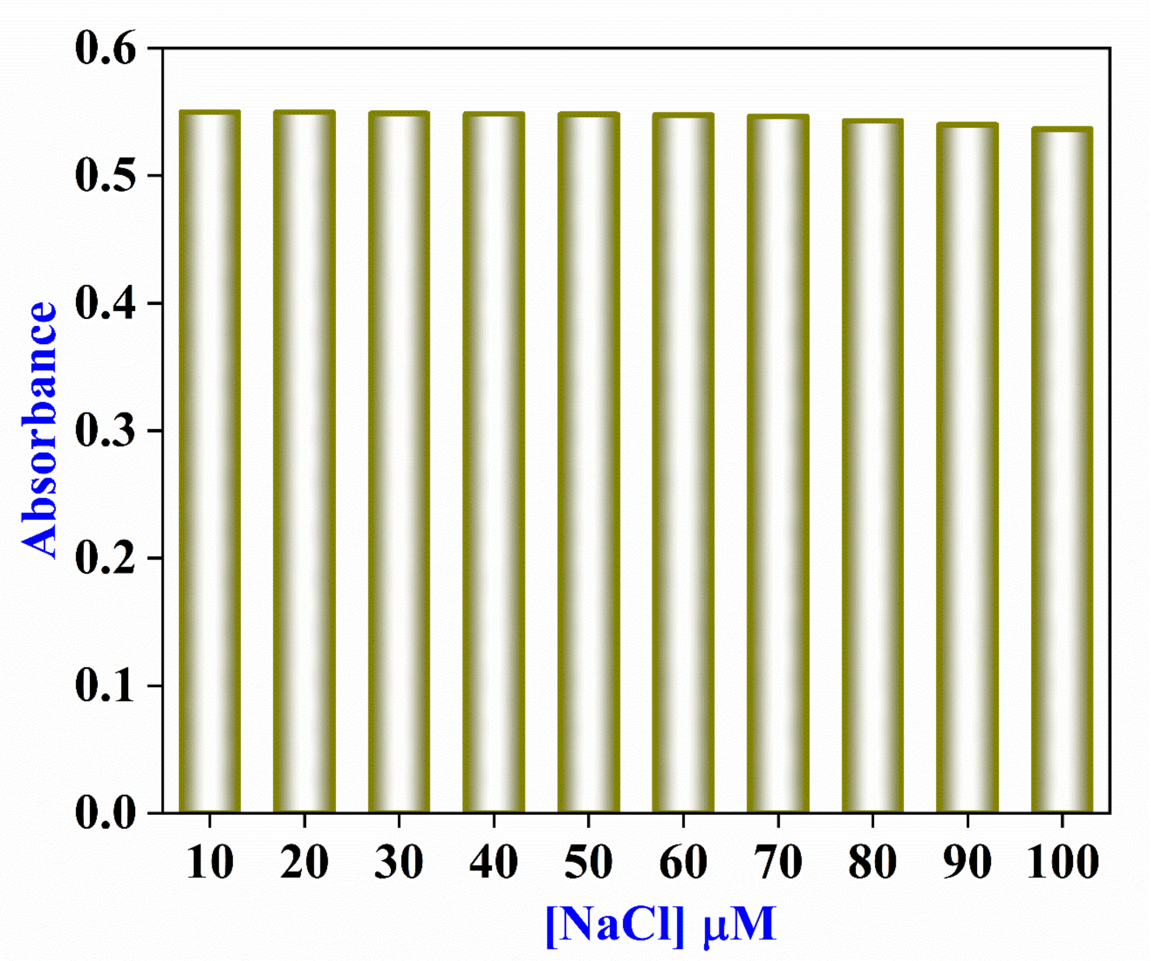

Figure S8. SPR band changes of β–CD-Ag/Au-rich alloy NPs with incremental amounts of NaCl. [NaCl] = 10-100 μM.

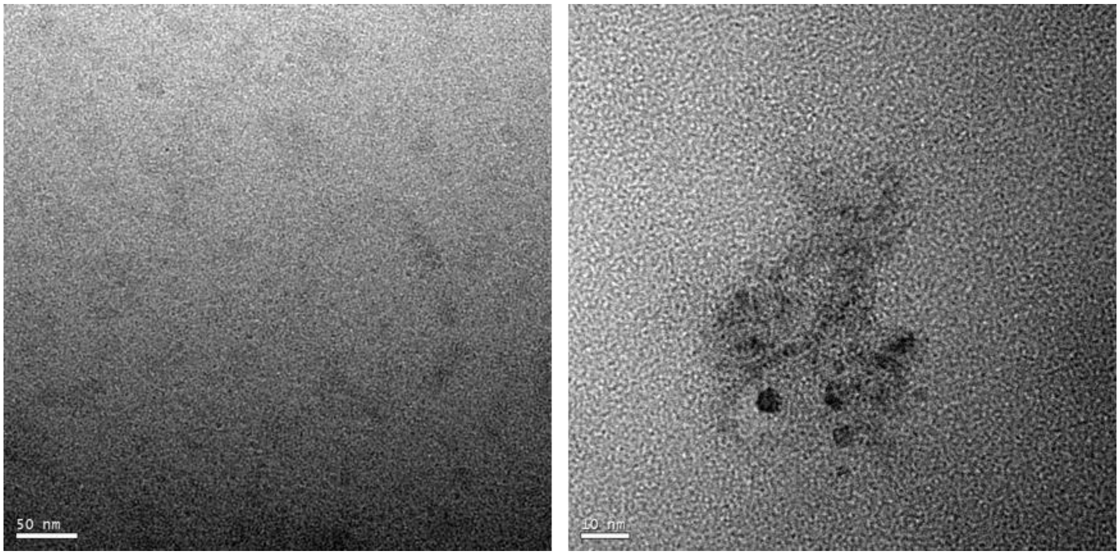

Figure S9. HR-TEM micrographs of β–CD-Ag/Au-rich alloy NPs with 60 nM of CN^{-} at different magnifications.

Table S1. EDX data for β−CD-Ag/Au-rich alloy NPs

Element	Line Type	k Factor	Wt%	Atomic %
Ag	K series	10.869	5.97	10.39
Au	L series	2.287	94.03	89.61
Total:			100.00	100.00

Table S2. RGB color variation results for β–CD-Ag/Au-rich alloy NPs with different quantities of CN^- ions

Concentrations (nM)	RED	GREEN	BLUE	R+G+B	R+G+B/R
Blank	190	65	0	255	1.342105
10	150	44	0	194	1.293333
20	123	40	15	178	1.447154
30	175	77	6	258	1.474286
40	154	70	27	251	1.62987
50	160	92	66	318	1.9875
60	158	99	77	334	2.113924
70	156	125	113	394	2.525641
80	159	137	129	425	2.672956
90	166	153	144	463	2.789157
100	168	164	154	486	2.892857

Table S3. Comparison between the analytical parameters of the present work with previously reported Au/Ag bimetallic nanostructures.

S. No	Method	Probe	Reaction time (min)	Linear range	LOD	Ref
1	Colorimetry with optical fiber sensor	Au_{core}-Ag_{shell} NPs	Not optimized	0-150 µM	0.08 nM	5
2	Colorimetry	Au@Ag core/shell nanoparticles	1	0.4-100 µM	0.4 µM	22
3	Colorimetry	Au@Au–Ag yolk–shell NPs	3	4-15 µM	---	23
4	Colorimetry	Ag@Aucore–shell NPs	Not optimized	0.4-32 µM	0.16 µM	26
5	Fluorescence	Ag@Au core−shell nanoparticle (NP)/iridium(III) complex	5	0.05-80 µM	0.036 µM	50
6	Colorimetry	Au@Ag Core/Shell Nanorods	1	1-200 µM	0.5 µM	51
**7**	**Colorimetry**<br>**Smartphone**	**β–CD-Ag/Au-rich alloy NPs**	**1**<br>**1**	**7.5-97.5 nM**<br>**10-100 nM**	**0.24**<br>**1.35**	**This work**

Table S4. Real water sample results for CN^- ions quantification based on β–CD-Ag/Au-rich alloy NPs by the SPR-based colorimetric platform and smartphone-based RGB color values tactics

Samples	CN^- ions spiked (nM)	CN^- ions found (nM)		Recovery (%)		RSD (n=5)	
		**SPR**	**RGB**	**SPR**	**RGB**	**SPR**	**RGB**
Drinking water	30.00	27.94	28.41	93.1	94.7	2.13	1.91
	60.00	58.64	57.97	97.3	96.6	1.81	1.54
	90.00	87.17	88.62	96.9	98.4	1.16	2.03
Tap water	30.00	28.23	28.15	94.1	93.8	2.98	3.07
	60.00	57.29	58.49	95.5	97.5	2.42	2.04
	90.00	86.87	87.36	96.5	97.1	2.31	1.84
Commercial drinking water	30.00	29.15	29.36	97.2	97.9	1.02	0.98
	60.00	58.65	58.79	97.8	98.0	1.62	1.01
	90.00	89.12	88.91	99.1	98.8	0.57	0.87

RSD= Relative standard deviation
